# Flash Glucose Monitoring to Assess Glycemic Control and Variability in Hemodialysis Patients: The GIOTTO Study

**DOI:** 10.3389/fmed.2021.617891

**Published:** 2021-07-30

**Authors:** Emanuele Mambelli, Stefania Cristino, Giovanni Mosconi, Christian Göbl, Andrea Tura

**Affiliations:** ^1^Nephrology and Dialysis, Bufalini Hospital, AUSL Romagna, Cesena, Italy; ^2^Nephrology and Dialysis, Morgagni-Pierantoni Hospital, AUSL Romagna, Forlì, Italy; ^3^Department of Obstetrics and Gynecology, Division of Obstetrics and Feto-Maternal Medicine, Medical University of Vienna, Vienna, Austria; ^4^CNR Institute of Neuroscience, Padova, Italy

**Keywords:** flash glucose monitoring, hemodialysis, glycemic variability, insulin sensitivity, insulin secretion, mathematical modeling

## Abstract

**Background:** Flash glucose monitoring (FGM) is a technology with considerable differences compared to continuous glucose monitoring (CGM), but it has been scarcely studied in hemodialysis patients. Thus, we aimed assessing the performance of FGM in such patients by comparison to self-monitoring of blood glucose (SMBG). We will also focus on estimation of glycemic control and variability, and their relationships with parameters of glucose homeostasis.

**Methods:** Thirty-one patients (20 with type 2 diabetes, T2DM, 11 diabetes-free, NODM) collected readings by FGM and SMBG for about 12 days on average. Readings by FGM and SMBG were compared by linear regression, Clarke error grid, and Bland-Altman analyses. Several indices of glycemic control and variability were computed. Ten patients also underwent oral glucose tolerance test (OGTT) for assessment of insulin sensitivity/resistance and insulin secretion/beta-cell function.

**Results:** Flash glucose monitoring and SMBG readings showed very good agreement in both T2DM and NODM (on average, 97 and 99% of readings during hemodialysis in A+B Clarke regions, respectively). Some glycemic control and variability indices were similar by FGM and SMBG (*p* = 0.06–0.9), whereas others were different (*p* = 0.0001–0.03). The majority of control and variability indices were higher in T2DM than in NODM, according to both FGM and SMBG (*p* = 0.0005–0.03). OGTT-based insulin secretion was inversely related to some variability indices according to FGM (*R* < −0.72, *p* < 0.02).

**Conclusions:** Based on our dataset, FGM appeared acceptable for glucose monitoring in hemodialysis patients, though partial disagreement with SMBG in glycemic control/variability assessment needs further investigations.

## Introduction

Flash glucose monitoring (FGM), based on the FreeStyle Libre system (Abbott Diabetes Care Inc.), is an interstitial glucose monitoring technology introduced in 2014, with some considerable differences compared to the more traditional continuous glucose monitoring (CGM) (1). As an example, a significant feature of the FGM system is that it is factory-calibrated, thus not requiring calibration by self-monitoring of blood glucose (SMBG) during the 14 days wearing period (2, 3), at difference with the majority of the CGM systems, especially those less recent. It is worth noting that, one the other hand, CGM has some advantages compared to FGM, especially the opportunity of some CGM systems to be reliably used in connection with an insulin pump, possibly automated (1). However, in patients not requiring such type of antidiabetic treatment, FGM option appears very interesting, even in the light of the typically lower costs when compared to the CGM systems.

Since its introduction, FGM has proven its clinical usefulness, as summarized in several review and meta-analysis studies (4–6). However, to our knowledge few studies analyzed the performance of the FGM system in patients undergoing chronic hemodialysis (7–9), and none assessed in details both glycemic control quality and glycemic variability.

Notably, even considering the more traditional CGM approach, relatively few studies focused on the assessment of glycemic control and variability in hemodialysis patients, and with limitations in the analysis, as well as, typically, in the studied patients' populations (10–16), despite the importance of glycemia assessment in hemodialysis has been clearly recognized (especially in the presence of type 2 diabetes) (17).

Specifically, during hemodialysis treatment, hyperglycemia may lead to hyponatremia, which can contribute to a wide spectrum of clinical symptoms, from mild to severe or even life threatening (18). The opposite risk for hemodialysis patients is hypoglycemia, which is not uncommon during hemodialysis even in patients without diabetes, and can even lead to coma or death (17). Of note, the risk for hypoglycemic events increases with intensive treatment, and in the presence of cardiovascular diseases it can cause fatal dysrhythmia (19). Moreover, it has to be recognized that high glycemic variability, compared to constant abnormal glycemia, may have even more dangerous cardiovascular effects, as mirrored by its high positive correlation with the urinary excretion rate of oxidative stress markers (20).

In this study, we aimed to assess the performance of the FGM system in hemodialysis patients, by comparison of FGM glucose readings to those obtained by SMBG, assumed as reference. We also focused on the estimation of both glycemic control and glycemic variability with the two approaches, in hemodialysis patients with type 2 diabetes and diabetes-free. In addition, we aimed to analyze in those patients possible relationships between glycemic control and variability and the main parameters of glucose homeostasis (mainly, insulin sensitivity/resistance, and insulin secretion/beta-cell function), as assessed by an oral glucose tolerance test (OGTT) performed in a subgroup of our study population.

## Materials and Methods

### Study Design, Participants, Data Collection

The present analysis considers data derived from the GIOTTO study (Glycemic patterns In patients undergOing chronic dialysis Treatment Through flash glucOse monitoring device). The study includes a cohort of patients undergoing chronic hemodialysis with and without type 2 diabetes, recruited at the Nephrology and Dialysis Units of the Bufalini Hospital (Cesena, Italy) and the Morgagni-Pierantoni Hospital (Forlì, Italy). Thirty-one patients were recruited (20 with type 2 diabetes, T2DM, and 11 without diabetes, NODM), undergoing hemodialysis three times per week from at least three months, with age in the 18–85 years range. For all patients dialysate composition contained 140 mmol/l sodium, 3 mmol/l potassium and 5.6 mmol/l glucose. Exclusion criteria were acute renal failure, pregnancy, transplantation requiring steroids treatment, inflammatory condition (acute or chronic), changes to the antidiabetic therapy during the study recruitment phase, neoplasia diagnosed in the last 5 years, psychological diseases, and any condition possibly affecting the patient's compliance to the study as determined by the investigators. Main patients' characteristics are reported in Table 1.

**Table 1:** Main characteristics of the patients, and number of glucose readings during the 14 days wearing period of the FGM sensor (mean ± SEM).

	**T2DM**	**NODM**
**Main characteristics**		
*N*	20	11
Sex (M/F)	12/8	10/1
Age (years)	63.3 ± 2.8	63.7 ± 4.5
BMI (kg/m^2^)	28.9 ± 1.5	22.8 ± 1.2
Hemodialysis duration (years)	3.4. ± 0.4	5.9 ± 1.4
Type 2 diabetes duration (years)	20.1 ± 1.8	–
Glycated hemoglobin (mmol/mol)	60.9 ± 4.5	36.6 ± 0.7
Fructosamine (μmol/l)	350.8 ± 15.0	283.6 ± 7.2
**Number of glucose readings**		
During hemodialysis sessions	23.3 ± 1.8	27.1 ± 1.2
In interdialytic periods	30.9 ± 3.2	–

A FGM sensor was applied to each patient to perform glucose readings for 14 days (according to the duration of the sensor). During every hemodialysis session, patients had to perform the glucose readings at the beginning of the session, and once per hour until the end of the session. Readings were collected by either the FGM reader or the personal smartphone (with the required application installed) as preferred. In order to collect pairs of glucose reading almost contemporary, with the same time scheduling (precisely, immediately before each FGM glucose reading), patients had to collect capillary blood glucose readings with traditional test strips method, i.e., SMBG. This was accomplished through Nova Pro glucometer system (Nova Biomedical, USA), which was chosen for some advantages compared to other glucose meters, such as the ability to eliminate interference with other electrochemical substances, as well as interference with abnormal hematocrit, thus providing very good results in terms of reliability. T2DM patients were also asked to continue with concomitant FGM and SMBG glucose readings in the interdialytic periods, with at least three readings per day.

During one of the hemodialysis sessions, a subgroup of 10 patients (five with and five without type 2 diabetes) underwent a 75 g OGTT (Glucosio Sclavo Diagnostics 75 g/150 ml, Doppel Farmaceutici, Italy), with measurement of plasma glucose, insulin and C-peptide at five time samples (0, 30, 60, 120, 180 min), performed at the AUSL Romagna Central Laboratory.

All patients provided written informed consent to the study, which was approved by the local ethics committee (n. 2616, 28/09/2017). The study was performed according to the guidelines of good clinical practice (ICH Harmonized Tripartite Guidelines for Good Clinical Practice 1996, Directive 91/507/EEC, and Italian D.M. 15/07/1997), and in agreement with the Helsinki declaration and with the Italian guidelines for conduction of clinical trials (D.L. n. 211, 24/06/2003, and D.M. 17/12/2004).

### Assessment of Glycemic Control and Glycemic Variability

The indices of glycemic control (21, 22) describe to what extent the glucose data tend to remain near a target value or in a target range. There are both basic indices of descriptive statistics, and more complex indices. As regards the former, we calculated the most common, i.e., the glucose mean (G_MEAN_), More complex and refined indices were the following (lower values mean better condition):

- GRADE (Glycemic Risk Assessment Diabetes Equation): glucose values are transformed to yield a continuous curvilinear response with minimum at about 5 mmol/l and high adverse weighting to hyperglycemia and hypoglycemia: GRADE = 425 × {log_10_[log_10_(G_*n*_)] + 0.16}^2^, G_*n*_ being the *n*-th glucose reading, in mmol/l; then, average value was taken;
- M-VALUE: it is a weighted average of the glucose values, with progressively larger penalties for more extreme values: M-VALUE = |10 × log_10_ (G_*n*_/IGV)|^3^, where IGV is the ideal glucose value, assumed equal to 120 mg/dl, as typically done; again, average value was then taken;
- LBGI (Low Blood Glucose Index): it is a transformation that normalizes the blood glucose scale: LBGI = 1.509 × {[log_e_(G_*n*_)]^1.084^ – 5.381}, for blood glucose values <112.5 mg/dl; then, a risk value is assigned to each blood glucose reading as follows: Risk(LBGI) = 10 × LBGI^2^; finally, average value was taken;
- High Blood Glucose Index (HBGI): similarly to LBGI, it is a transformation to normalize the blood glucose scale, for blood glucose values higher than 112.5 mg/dl; the expression of HBGI is the same as for LBGI;
- Average Daily Risk Range (ADRR): it is the sum of LBGI and HBGI, calculated with the minimum and the maximum glucose value, respectively.

The indices of glycemic variability (21, 22) measure to what extent the glucose data tend to oscillate: the higher the variability, the higher the value of the indices. Some basic indices that we calculated were the glucose standard deviation (G_SD_) and the total glucose range of variation (G_RANGE_). More refined indices were:

- J-INDEX: it is an index somehow intermediate between glycemic control and variability, as it is a combination of information from G_MEAN_ and G_SD_, but to our knowledge it is typically classified as a glycemic variability index: J-INDEX = 0.001 × (G_MEAN_+G_SD_)^2^;
- CONGA (Continuous Overlapping Net Glycemic Action): it is the standard deviation (SD) of the difference between values typically obtained 60 min apart, but in this case we performed the analysis over the glucose data available, though the time interval between consecutive values was not constant and typically higher than 1 h;
- MAGE (Mean Amplitude of Glycemic Excursion): it is the mean of the glycemic excursions that are >1 *SD*;
- Autocorrelation, AUTCORR: it considers to what extent the glucose values tend to repeat or change during time; the autocorrelation sequence is computed as $\text{Ad}(m)=\frac{1}{N-|m|}\sum_{i=1}^{N-|m|}x(i)\;\cdot \;x(i+m)$ where *x* in this case is glucose, *N* is the total number of samples and *m* is the time lag expressed as number of samples; then the sequence is normalized to Ad (1), and average value is calculated to get the autocorrelation index.

The indicated indices were calculated separately on SMBG and FGM values for each patient. For both SMBG and FGM values, we calculated the indices over the whole patient's data and, for T2DM patients, separately over the data collected during the hemodialysis sessions and during the interdialytic periods.

### Assessment of Glucose Homeostasis From the OGTT

Modeling approach was used for the assessment of pancreatic insulin secretion and beta-cell function (23, 24). Briefly, the main beta-cell function parameters were beta-cell glucose sensitivity, G_SENS_ (mean value of the dose-response function), describing the dependence of insulin secretion on absolute glucose concentration; rate sensitivity, R_SENS_ (proportionality constant of the derivative component of the secretion), representing the dynamic dependence of secretion on the rate of change of glucose; ratio of the potentiation factor at 180 to that at 0 min, PFR, explaining the sustained insulin secretion levels often seen at the end of an OGTT even if glucose has already returned to basal (partly related to the enhancing effect of incretin hormones on insulin secretion). Basal and total insulin secretion were also computed (ISR_b_ and ISR_t_, respectively).

Insulin sensitivity/resistance was estimated at fasting by the homeostasis model assessment—insulin resistance index, HOMA-IR (25), and during the OGTT by the recently developed predicted M index, PREDIM (26).

### Statistical Analysis

Comparison of glucose readings derived by SMBG and FGM was performed according to linear regression and Clarke error grid analyses. We also reported Bland-Altman plots.

The indices of glycemic control and glycemic variability obtained by SMBG and FGM were compared by paired *t*-test. Unpaired *t*-test was used to compare the indices between T2DM and NODM patients, obtained by both SMBG and FGM.

By linear regression analysis we also analyzed possible relationships among glycemic control/variability indices and glucose homeostasis parameters.

Normality of distribution of the analyzed indices and parameters values was tested with the Shapiro-Wilk test. In the case of skewed distribution, values were logarithmically transformed before performing the indicated statistical testing. For the unpaired *t*-test, in case of inhomogeneity of parameters variances as assessed with Levene test, appropriate correction was performed (not-pooled standard deviation correction).

Values are presented as mean ± standard error of the mean (SEM), unless otherwise specified. Two-sided *p* < 0.05 was considered as statistically significant. Statistical analyses were performed in R (version 3.6.1).

## Results

### FGM Sensor Wearing and Number of Glucose Readings

T2DM collected pairs of concomitant glucose readings (i.e., by both SMBG and FGM) on average for 12.5 ± 0.6 days, and similarly for NODM (12.0 ± 0.6 days, though with readings only during hemodialysis sessions). All patients collected readings for at least 7 days, except for one T2DM patient (3 days, with only interdialytic readings). Typical reason for stopping readings was FGM sensor accidental detachment, though some patients in that event requested to continue by wearing a new sensor (one T2DM patient thus collecting readings for 16 days). Information on the number of glucose readings, collected by SMBG and FGM in the two groups, are reported in Table 1.

### Comparison of SMBG and FGM Glucose Readings

We first compared the glucose readings by SMBG and FGM in each patient by linear regression analysis. During the hemodialysis sessions, in T2DM, apart for the patient lacking readings, only one patient showed not significant relationship between SMBG and FGM (*p* = 0.16), probably due to the low number of readings (8 readings only). In two patients, the relationship was significant though not strong (*R* = 0.37 and 0.49, with *p* = 0.04 and 0.01, respectively). In the other 16 patients the relationship was strong, or even extremely strong (*R* = 0.66–0.97, with p always <0.0001). In NODM, the relationship was not significant in two patients (*p* > 0.13), significant but not strong in one patient (*R* = 0.40, *p* = 0.03), and typically strong in the other eight patients (*R* = 0.52–0.72, *p* < 0.01, until *p* < 0.0001 in some patients). When considering the readings of all patients grouped, the relationship between SMBG and FGM showed *R* = 0.77, *p* < 0.0001 (*R* = 0.79, *p* < 0.0001, when excluding the three not significant cases).

In the interdialytic periods, readings were available only for T2DM. We found no significant relationship in two patients (*p* > 0.3), whereas the relationship was strong or very strong in the other patients (*R* = 0.63–0.98, *p* < 0.008, until *p* < 0.0001). In all T2DM patients grouped, we found *R* = 0.80, *p* < 0.0001 (*R* = 0.83, *p* < 0.0001, when excluding not significant cases).

With dialytic and interdialytic readings grouped, in T2DM the relationship between SMBG and FGM was significant for all patients: not strong in one patient only (*R* = 0.30, *p* = 0.04), and strong or very strong in the other 19 patients (*R* = 0.61–0.97, *p* < 0.008, until *p* < 0.0001).

When considering all readings of all patients, including both T2DM and NODM, the relationship between SMBG and FGM showed *R* = 0.80, *p* < 0.0001 (*R* = 0.81, *p* < 0.0001, excluding not significant cases).

**Figure 1:**
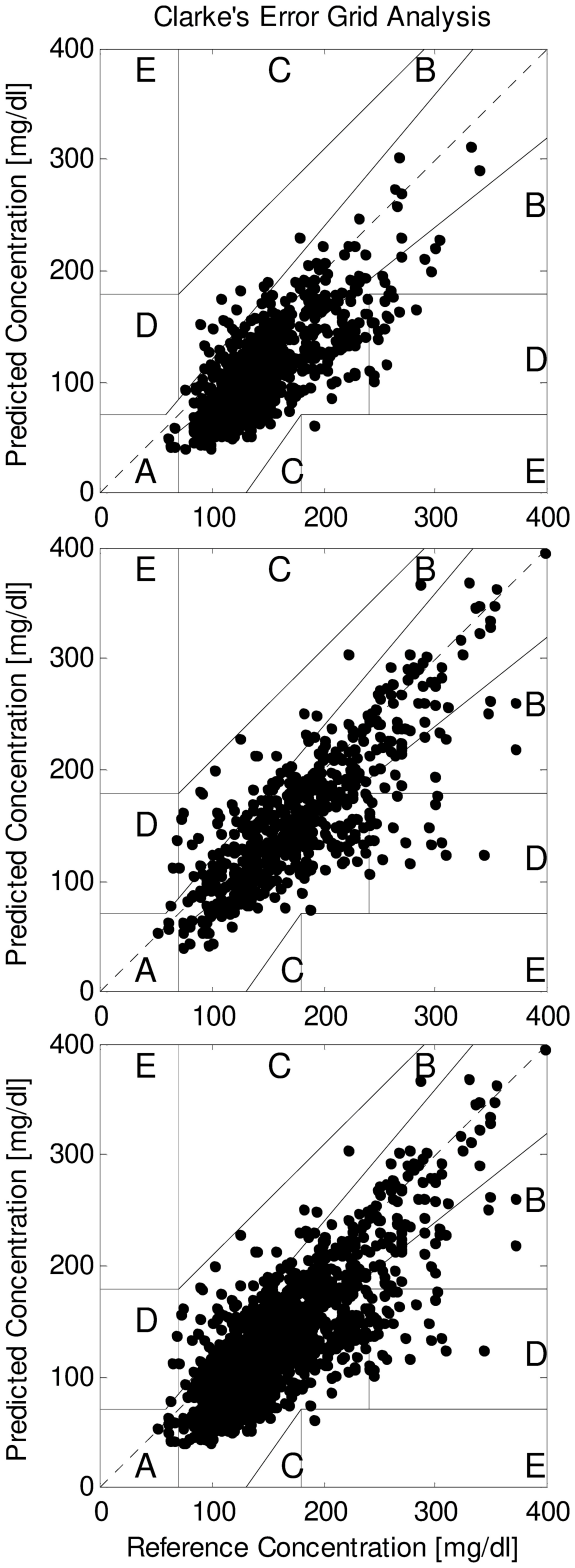
Clarke error grid analysis of SMBG and FGM readings (reference and predicted, respectively) in all patients grouped, for readings during hemodialysis sessions (upper panel), during interdialytic periods (only T2DM patients; intermediate panel), and for all readings grouped (lower panel).

We also compared SMBG and FGM readings by Clarke error grid analysis. During the hemodialysis sessions, in T2DM the number of readings in A region ranged from a minimum of 3.4% to a maximum of 100%, with average equal to 41.8%. When considering A+B regions, the agreement between SMBG and FGM increased to 97.0% on average, with minimum of 80.0%. In NODM, readings in A region were on average 36.0% (7.1–75.0% range), whereas readings in A+B regions increased to 99.3% on average (93.1–100% range). With all patients grouped, readings in A region were 39.8%, and 97.8% in A+B region (Figure 1, upper panel).

In the interdialytic periods, in T2DM the readings in A region were on average 57.8% (4.3–100% range); in A+B regions, readings were on average 96.0%, with minimum of 80.0%. With all T2DM patients grouped, readings in A region were 59.5%, and 96.1% in A+B region (Figure 1, intermediate panel).

With all readings grouped, in T2DM the readings in A region were on average 51.2% (11.1–92.5% range), whereas in the A+B regions they were on average 96.6% (85.5–100% range).

When considering all readings in all patients, readings in A region were 45.7%, and 97.6% in A+B regions (Figure 1, lower panel).

**Figure 2:**
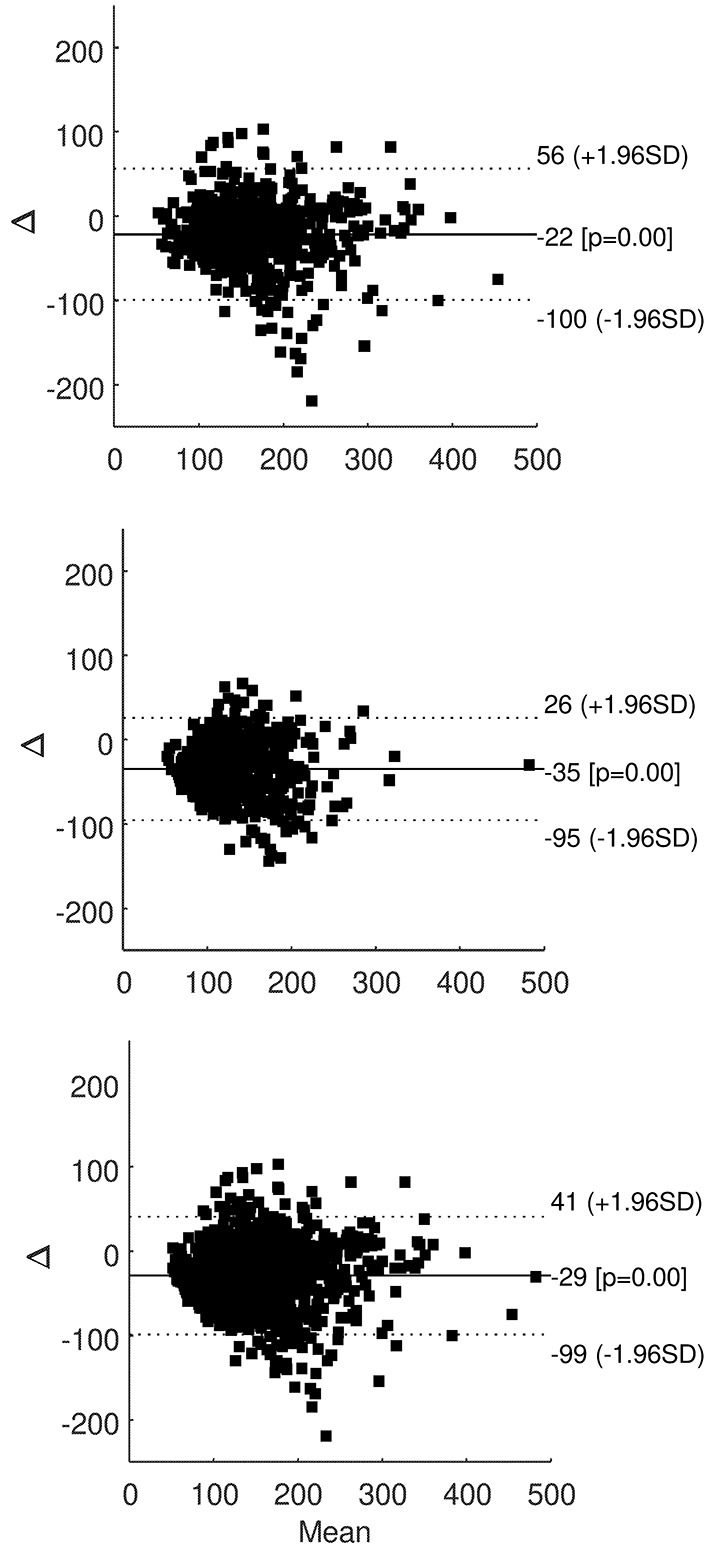
Bland-Altman plot of SMBG and FGM readings in all patients grouped, for readings during hemodialysis sessions (upper panel), during interdialytic periods (only T2DM patients; intermediate panel), and for all readings grouped (lower panel); solid line is average difference (with *p*-value compared to zero difference), dotted lines are ±1.96 standard deviation (*SD*) of the difference.

When performing Bland-Altman plots, in each patient the majority of samples fell within the ±1.96 standard deviation limits of agreement. Plots for all patients grouped are reported in Figure 2, showing the great majority of samples within the limits of agreement both during hemodialysis sessions (upper panel) and in interdialytic periods (intermediate panel), and with all glucose readings grouped (lower panel).

### Glycemic Control and Glycemic Variability

We calculated the indices of glycemic control and glycemic variability based on both SMBG and FGM readings (Table 2).

**Table 2:** Indices of glycemic control and glycemic variability derived by SMBG and FGM (upper and lower, respectively) in T2DM patients during hemodialysis sessions (HD), during interdialytic periods (INTERD), and with all values; in NODM patients, during HD (mean ± SEM).

	**T2DM in HD**	**T2DM in INTERD**	**T2DM (all)**	**NODM in HD**
**Glycemic control**				
G_MEAN_ (mg/dl)	161.59 ± 6.65	183.84 ± 8.15	174.43 ± 6.47	126.82 ± 4.16
	127.00 ± 7.38	163.35 ± 9.23	149.40 ± 8.95	96.69 ± 4.81
GRADE (unitless)	1.70 ± 0.24	2.86 ± 0.31	2.37 ± 0.26	0.67 ± 0.10
	2.89 ± 0.60	2.71 ± 0.36	2.94 ± 0.39	4.14 ± 0.91
M-VALUE (unitless)	7.93 ± 1.78	15.51 ± 2.44	12.38 ± 2.01	1.35 ± 0.54
	8.08 ± 1.85	12.44 ± 2.60	11.44 ± 2.30	9.01 ± 2.32
LBGI (unitless)	0.19 ± 0.09	0.32 ± 0.13	0.26 ± 0.08	0.31 ± 0.08
	2.39 ± 0.71	0.91 ± 0.27	1.61 ± 0.41	4.44 ± 1.04
HBGI (unitless)	6.50 ± 1.04	10.70 ± 1.39	8.94 ± 1.11	1.54 ± 0.49
	3.12 ± 0.77	7.83 ± 1.53	6.21 ± 1.35	0.46 ± 0.15
ADRR (unitless)	26.14 ± 3.91	40.39 ± 4.09	42.96 ± 4.48	9.75 ± 2.20
	27.93 ± 4.23	42.22 ± 4.50	47.37 ± 4.67	23.05 ± 3.26
**Glycemic variability**				
G_SD_ (mg/dl)	41.20 ± 4.26	52.60 ± 4.00	52.26 ± 3.95	23.26 ± 2.97
	38.21 ± 4.33	49.59 ± 3.97	50.02 ± 3.87	25.84 ± 2.13
G_RANGE_ (mg/dl)	153.58 ± 16.38	210.05 ± 17.01	231.00 ± 19.46	83.36 ± 10.32
	145.05 ± 18.71	198.55 ± 15.61	220.25 ± 18.54	96.36 ± 7.08
J-INDEX (10^−3^(mg/dl)^2^)	42.92 ± 4.40	58.14 ± 5.09	53.11 ± 4.41	22.96 ± 2.20
	29.37 ± 4.08	47.94 ± 5.40	42.32 ± 5.14	15.38 ± 1.46
CONGA (mg/dl)	47.59 ± 5.87	73.76 ± 6.07	64.39 ± 4.95	30.11 ± 5.58
	39.81 ± 6.29	67.84 ± 6.03	57.83 ± 4.94	27.38 ± 2.93
MAGE (mg/dl)	93.74 ± 13.21	108.23 ± 9.43	112.15 ± 9.72	47.88 ± 6.95
	85.59 ± 13.67	104.48 ± 8.72	108.70 ± 9.32	52.16 ± 3.74
AUTCORR (unitless)	0.33 ± 0.02	0.32 ± 0.02	0.23 ± 0.01	0.34 ± 0.03
	0.38 ± 0.03	0.34 ± 0.03	0.28 ± 0.03	0.44 ± 0.05

In T2DM during hemodialysis, G_MEAN_ was significantly higher when assessed by SMBG (*p* < 0.0001), but the more refined indices of glycemic control (GRADE and M-VALUE) were not significantly different (*p* = 0.1 and *p* = 0.9, respectively). When looking to indices focused on low (LBGI) and high glucose values (HBGI), we found again significant differences between SMBG and FGM assessment (*p* < 0.0003). However, when considering the combination of LBGI and HBGI (i.e., ADRR), SMBG and FGM provided similar values (*p* = 0.33). With regard to glycemic variability, G_SD_ was slightly different when assessed by SMBG and FGM (*p* < 0.03), but G_RANGE_ was not different (*p* = 0.21). More refined indices showed somehow contrasting behavior, as J-INDEX and CONGA were different (*p* < 0.004), whereas MAGE and AUTCORR were not, though the former with borderline *p*-value (*p* = 0.055 and *p* = 0.08, respectively).

In T2DM in the interdialytic periods, for glycemic control results were similar to those during the hemodialysis sessions: G_MEAN_ was different between SMBG and FGM assessment (*p* < 0.006), but GRADE and M-VALUE were not (*p* > 0.09). Again, LBGI and HBGI were different (*p* < 0.002), but not ADRR (*p* = 0.57). For the glycemic variability, all indices were not different (with *p* ranging from 0.07 to 0.65), except for J-INDEX (*p* < 0.008).

When considering all glycemic readings in T2DM, glycemic control indices confirmed what already reported (G_MEAN_ different, but not GRADE and M-VALUE, and LBGI/HBGI different, but not ADRR). For glycemic variability, J-INDEX and CONGA were different, whereas all other indices (G_SD_, G_RANGE_, MAGE, AUTCORR) were not.

In NODM during hemodialysis sessions, we found lack of agreement between SMBG and FGM for the indices of glycemic control (*p* < 0.02), whereas in contrast the indices of glycemic variability were not different (*p* > 0.1), except for J-INDEX (*p* = 0.0003), probably due to the effect of G_MEAN_ on the index values.

We then compared the indices of glycemic control and variability between T2DM and NODM during hemodialysis sessions (NODM lacking glucose readings in interdialytic periods). Indices of glycemic control assessed by SMBG values were higher in T2DM than in NODM (*p* < 0.0007), except for LBGI (*p* = 0.15). Indices assessed by FGM showed similar behavior for G_MEAN_ (*p* = 0.007) and HBGI (*p* = 0.03), whereas other indices did not reach statistical significance (*p* > 0.06), though LBGI and ADRR showed similar tendencies to the corresponding indices assessed by SMBG.

Indices of glycemic variability assessed by SMBG were higher in T2DM (*p* < 0.03), except for AUTCORR (*p* = 0.68). Indices assessed by FGM essentially confirmed the SMBG-based results (*p* < 0.03), with only CONGA not reaching statistical significance (*p* = 0.10).

Notably, when all glucose readings are considered in T2DM, according to SMBG all indices of glycemic control and variability were different than in NODM (*p* < 0.0005), and FGM showed excellent agreement [significant difference for all indices of glycemic variability and four indices of glycemic control (*p* < 0.007), with only GRADE and M-VALUE not different (*p* > 0.17)].

### OGTT-Derived Parameters and Relationships With the Indices of Glycemic Control and Variability

Table 3 reports the values of the OGTT-derived parameters, with indication of possible relationships with the indices of glycemic control and variability (assessed by glucose readings during hemodialysis sessions only, or by all glucose readings).

**Table 3:** OGTT-derived parameters (mean ± SEM), and their relationships with the indices of glycemic control (GC) and variability (GV).

		**Relationship with GC**	**Relationship with GV**
**Beta-cell function and insulin secretion**			
G_SENS_ (pmol min^−1^ m^−2^ mM^−1^)	35.84 ± 10.90	Uncertain	Uncertain
R_SENS_ (pmol m^−2^ mM^−1^)	662.54 ± 503.42	Uncertain	No
PFR (unitless)	0.88 ± 0.07	Uncertain	No
ISR_b_ (pmol min^−1^ m^−2^)	444.35 ± 51.11	Uncertain	No
ISR_t_ (nmol m^−2^)	94.77 ± 12.06	Uncertain	Yes
**Insulin sensitivity/resistance**			
HOMA-IR (unitless)	4.77 ± 2.50	No	No
PREDIM (mg kg^−1^ min^−1^)	3.65 ± 0.33	No	No

*No, no relationship with any index; Uncertain, relationship with at least one index; Yes, relationship with two or more indices*.

**Figure 3:**
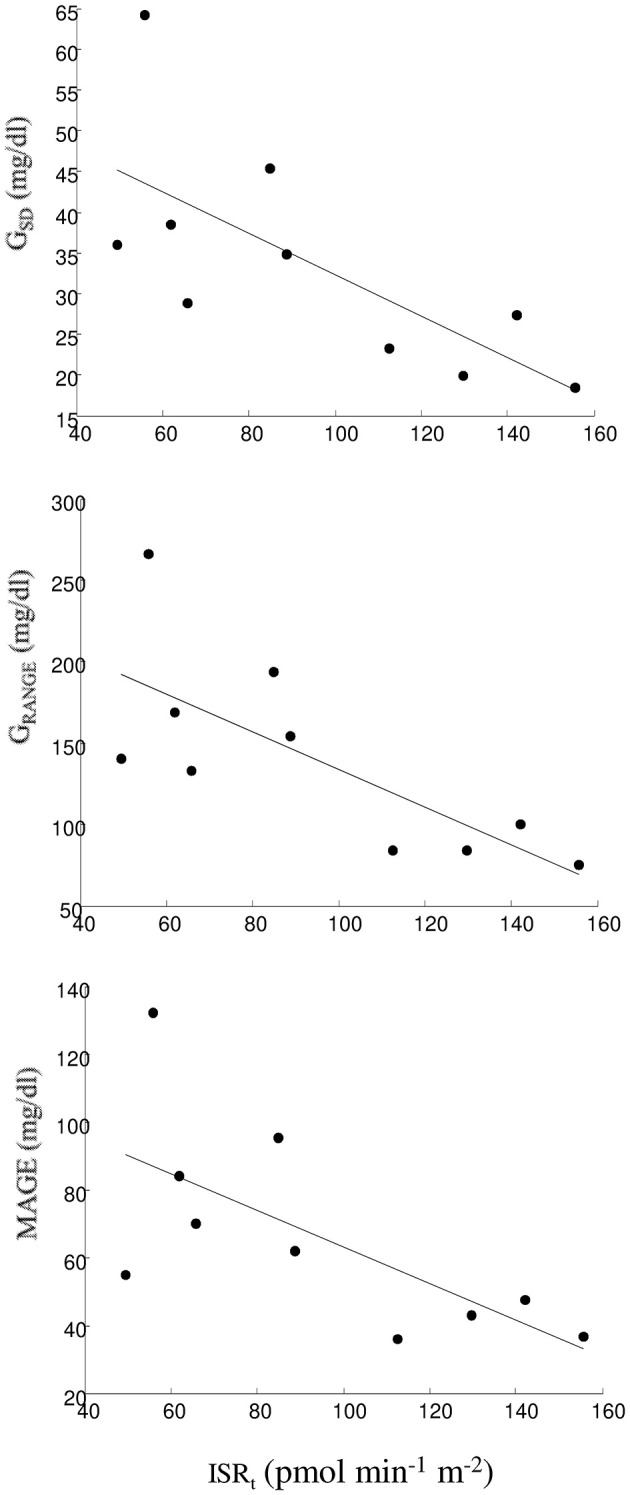
Linear regression plot between ISR_t_ and G_SD_ (upper panel), G_RANGE_ (intermediate panel), and MAGE (lower panel).

Relationship between an OGTT parameter and the indices of glycemic control and variability was assumed as uncertain if at least one relationship was found significant, and it was assumed as present in the case of significant relationship with two or more indices, separately for the indices of glycemic control and glycemic variability (considering the indices computed from at least one between SMBG and FGM; Table 3). Generally, few relationships were found significant. The most evident relationship was observed between total insulin secretion, ISR_t_, and glycemic variability, with significant inverse relationship with G_SD_, G_RANGE_, and MAGE, showing relatively high *R*-value, ranging from −0.72 to −0.79 with *p* from 0.02 to 0.007 (Figure 3). Notably, these three significant relationships were found with the indices computed by FGM.

## Discussion

In this study, we assessed the performance of the FreeStyle Libre FGM system in hemodialysis patients, by comparison to glucose readings obtained by SMBG. In fact, it is well known that there may be a time lag between glucose readings in blood and in the interstitial fluid (27). In addition, hemodialysis patients may undergo volume changes among the intracellular, interstitial, and intravascular compartments, especially during hemodialysis sessions (28), but partially also in the interdialytic periods due to factors such as systemic inflammation, endothelial dysfunction, hypoalbuminemia, and increased capillary permeability, leading to intravascular fluid overload (29). These phenomena may further affect the possible difference between glucose measurement in blood and in the interstitial fluid. Thus, a detailed analysis of the FGM performance compared to SMBG appeared appropriate.

In our study, we found very good agreement between SMBG and FGM, as assessed by linear regression, Clarke error grid and Bland-Altman analyses. In the Clarke error grid analyses, in all patients grouped and considering both hemodialysis and intradialytic readings, 97.6% of the readings fell within the A+B regions, i.e., within the regions typically considered as clinical acceptable (30). Results essentially similar (even slightly better) were found with Parkes error grid analysis (31), with 98.6% of all readings in A+B regions (53.2% in A, 45.4% in B), the remaining 1.4% in C region, and none in D or E regions (details not shown). It is also worth noting that, with regard to regression analysis over all patients grouped, we also performed adjustment for repeated measures (to account for the several readings in each patients) by calculation of the marginal coefficient of determination for generalized mixed-effect models (that is, a pseudo-R-squared value), and found however results very similar to those of the traditional linear regression (details not shown).

Based on SMBG and FGM readings, we also computed several indices of glycemic control and variability. Some indices were not significantly different when computed with either SMBG or FGM, whereas others showed lack of agreement. Especially, more considerable differences were found for the basic indices rather than the more refined ones, this suggesting that such type of analyses should not be limited to the basic indices. On the other hand, it should be acknowledged that, when computing several indices, some may be interrelated (i.e., not statistically independent), and hence caution should be used when including them in some types of statistical analyses that may suffer for problems of multicollinearity among variables. This possible drawback was however not applicable to the analyses performed in our study.

It should also be observed that somehow greater lack of agreement was observed in NODM patients. This may be due to the reason that these patients typically do not perform SMBG, thus some inaccuracies in the SMBG readings may have derived by not totally appropriate execution of such readings. It is known in fact that some patient's related factors may affect the accuracy of SMBG (32), though it should be observed that patients were typically helped to perform the readings during hemodialysis, thus possible inaccuracies may be due to other factors. At any rate, some degree of inaccuracy in the glucose readings may have influenced the results of our comparisons, especially when comparing the glycemic control and variability indices rather than the single glucose readings, due to the much lower number of samples available for comparison in the first case. In spite of this, when comparing glycemic control and variability indices between T2DM and NODM, we found substantial agreement between results obtained by SMBG and FGM. In future studies we plan to investigate in details the reasons for the reported partial disagreement observed particularly in NODM patients. Specifically, an aspect that needs further analysis is the range of variation of the time lag between the interstitial and blood glucose concentrations.

In a subgroup of patients that agreed to undergo an OGTT during one of the hemodialysis sessions, we analyzed possible relationships between glycemic control and variability indices with the main parameters of glucose homeostasis (insulin sensitivity/resistance, and pancreatic insulin secretion/beta-cell function). Of note, we calculated insulin sensitivity/resistance both at fasting (insulin resistance: HOMA-IR) and during the OGTT (insulin sensitivity: PREDIM), since they may describe somehow different physiological processes. In fact, it has been reported that fasting insulin resistance may particularly reflect hepatic insulin resistance, whereas OGTT-based insulin sensitivity may be mainly related to peripheral insulin sensitivity (33).

The majority of the relationships between glycemic control and variability indices and the OGTT parameters was found as not significant, but we observed a marked inverse relationship between total insulin secretion and some indices of glycemic variability. Few studies provided overall analyses of glucose homeostasis in hemodialysis patients, with special attention to both insulin sensitivity/resistance and beta-cell function, and to our knowledge none explored the possible relationships between glycemic control/variability and the parameters of glucose homeostasis. However, our findings appear consistent with some previous studies in different populations (i.e., not with renal diseases) where similar inverse relationship between glycemic variability and insulin secretion/beta-cell function was observed, likely due to the fact that the fluctuations of blood glucose can lead to deterioration of the beta-cell function (possibly due to beta-cell apoptosis caused by damage to cellular defense homeostasis), though on the other hand an increase in glucose fluctuations may also be a consequence of defective beta-cell function (34–38).

To our knowledge, very few studies used FGM in patients undergoing hemodialysis (7–9, 39). In the first of those studies (7), 32 patients, both with and without diabetes, were studied. However, FGM performance was not assessed in comparison to another methodology for glucose measurement. Furthermore, the analysis of both glycemic control and variability was extremely limited. In the second of the indicated studies (8), ten hemodialysis patients with type 2 diabetes were studied, whereas non-diabetic patients were not included; also, the analysis of the FGM readings was limited to 24 h only, and again without any comparison to glucose readings obtained with another approach. Furthermore, similarly to the previously indicated study (7), the assessment of glycemic control and variability was limited. Similar limitations hold also for the other two indicated studies (9, 39), with regard to glucose readings duration and study population (thirteen patients with type 2 diabetes, again without non-diabetic patients), though in these studies it has to be appreciated the concomitant use of both FGM and CGM systems, in addition to SMBG monitoring. In our study, CGM system was not used, for reasons of project cost containment, and to limit the burden and discomfort of the study to the patients. In another study (40), FGM was used in 18 hemodialysis patients with type 2 diabetes, but the focus was on glycated hemoglobin, as FGM readings were used to compute an estimated glycated hemoglobin value (eHbA1c) to be compared with eHbA1c derived by glycated albumin, BMI, and hemoglobin.

Of note, even considering the use of the CGM approach (i.e., no FGM), a relatively limited number of studies focused on the assessment of both glycemic control and variability in hemodialysis patients (10–16). In the first of those studies (10), glycemic variability was found higher in patients with type 2 diabetes compared to non-diabetic patients (in agreement with our findings), though analysis was limited to 72 h. Similar limitation holds for the second of the indicated studies (11) (48 h of monitoring), and in addition only patients with type 2 diabetes were studied. The following two studies (12, 13), similarly included only patients with diabetes, though CGM was performed both during hemodialysis sessions and in the interdialytic periods. In the last three studies (14–16), CGM was performed with the main purpose to assess the effect on glycemia of pharmacological interventions, with population again limited to patients with diabetes. It should also be noted that all these studies did not report detailed analysis of glycemic control and variability, often presenting basic indices and only rarely some more refined indices (typically, MAGE). In some other studies glycemic control/variability was assessed by traditional methodologies (i.e., neither CGM nor FGM) (41–45), though again with the indicated limitations in the analysis. One study (46) reported glycemic variability information over a long time period, but with extremely sparse data; it was found that higher glycemic variability is associated with increased mortality risk in patients with diabetes undergoing hemodialysis.

In our study, we calculated several indices of both glycemic control and variability. Nevertheless, it should be acknowledged that many other indices may be computed [as reported, for instance, in studies (21, 22)]. However, for brevity and hence better readability, as well as to limit possible redundancy, we selected for the analyses those indices that, to our knowledge, are more common or more informative, also based on some of our previous studies (47–49).

The study has some limitations. First, the number of patients included in the study was not large, but this mainly depends on the difficulty in identifying appropriate subjects, i.e., hemodialysis patients matching all the inclusion criteria and willing to participate to the study. It should also be noted that the number of patients of our study was nonetheless typically higher than that of previous relevant studies in the field (7–16, 39), with only a couple of studies having similar or slightly higher number of participants [i.e., 38 and 46 subjects (7, 10)].

In addition, it should be acknowledged that not all patients collected the same number of glucose readings, but this is expected in such kind of studies. Also, in some occasions, consecutive glucose readings were separated by a considerable time interval (particularly in the interdialytic periods), and this is not optimal condition for the assessment of glycemic control and, especially, glycemic variability. Furthermore, NODM patients collected glucose readings only during the hemodialysis sessions, but this was due to the reason that, when preparing the study protocol, investigators agreed that in NODM patients very poor compliance may have been obtained for SMBG outside the Hospital.

It should also be acknowledged that glucose readings in blood were obtained by SMBG and not by laboratory glucose analyzer. However, since we collected glucose readings not only during hemodialysis sessions (i.e., at the Hospital), but also during the interdialytic periods (at least in T2DM patients), SMBG was a necessary choice.

As regards the OGTT, a first limitation was the lack of overnight fasting before its execution. Indeed, the majority of the hemodialysis sessions (OGTT was performed during one of them) were programmed in the afternoon, and hence it was unfeasible asking for totally fasting condition. Nonetheless, it was asked not to eat or drink in the 3 h before the hemodialysis plus OGTT session. Notably, in NODM patients, when inspecting glycemic values at 0 min of the OGTT (i.e., immediately before the glucose ingestion), we found on average 89.6 mg/dl, and not exceeding 100 mg/dl. On the other hand, glycemic values below 100 mg/dl identify normal glucose tolerance in fasting condition (50). Thus, the low glycemic values observed in NODM after at least the three hours of fasting may suggest that in our hemodialysis patients a condition similar to actual fasting was reached before the OGTT.

Another limitation was related to the assessment of the OGTT-based insulin secretion and beta-cell function. In fact, this requires the knowledge of the C-peptide kinetics (including C-peptide clearance), which is obtained by the method of Van Cauter et al. through simple patient's information (such as anthropometric data) (51). However, since C-peptide is mainly cleared by the kidneys (52), in patients with end-stage renal disease the method of the study indicated above (51) may not be totally accurate. On the other hand, to our knowledge there is no method to derive information on C-peptide kinetics specific for patients with end-stage renal disease, unless extremely invasive approaches are used. Furthermore, it should be noted that performing the OGTT during a hemodialysis session should have limited the indicated problem, as in that period the kidneys function is at least partially replaced by the treatment.

In the OGTT, possible confounding factor may also be the presence of glucose in the dialysate, this having advantages and disadvantages. In fact, study (53) reported that some decades ago dialysate glucose was used even at extremely high concentration values (up to 100 mmol/l), since osmotic ultrafiltration (with sodium and glucose being the major osmoles) was the main mode of volume removal. Subsequently, ultrafiltration by hydrostatic pressure was found superior to osmotic ultrafiltration and the concentration of dialysate glucose was decreased. Indeed, concerns in using glucose in the dialysate included hypertriglyceridemia, risk for less effective potassium removal, and possible bacterial/fungal growth in the dialysate. In addition, hyperglycemia can activate inflammatory pathways by different mechanisms. Glucose can react with several substrates to form advanced glycosylation end-products that subsequently lead to oxidative stress and activation of proinflammatory cytokines (54), and this has been suggested to be linked with increased mortality in end-stage renal disease patients (55). On the other hand, glucose-free dialysate determines risk for hypoglycemia (especially in diabetic patients taking insulin) and greater amino acid losses due to induction of a catabolic state (53). It was also reported that glucose-added dialysate was superior to glucose-free dialysate in the protection of the central nervous system of hemodialysis patients (56). Thus, in our study we used glucose-added dialysate, though not at high concentration (5.6 mmol/l), as a compromise among the discussed advantages and disadvantages of dialysate glucose. Of course, this can have affected the plasma glucose concentration values measured during the OGTT, thus leading to possible bias in the calculation of some glucometabolic parameters. On the other hand, since the dialysate glucose was the same in all patients, we expect that the main results of the OGTT analysis were not strongly affected.

In conclusion, in the present study we have used the FreeStyle Libre FGM monitoring system in patients undergoing chronic hemodialysis treatment, both with and without type 2 diabetes. To our knowledge, this is the first deep analysis of FGM performance in hemodialysis patients with diabetes and diabetes-free. When compared to SMBG readings taken as reference, we found very good agreement in the glucose readings obtained by the two approaches, both during the hemodialysis sessions and in the interdialytic periods. Thus, based on our dataset, we suggest that FGM should be adequate for glucose monitoring in patients undergoing chronic hemodialysis. We also aimed to evaluate glycemic control and variability, whose importance in hemodialysis patients has been clearly established (especially in the presence of type 2 diabetes), and to our knowledge this is the first study in hemodialysis patients with detailed assessment of both these glycemic aspects. We found that some indices of glycemic control and variability provided similar information when computed by SMBG and FGM readings, whereas others revealed some lack of agreement. Future studies should investigate the reason for such possible disagreement. However, when comparing T2DM to NODM patients, as expected we observed in the former higher values for both glycemic control and variability indices. Finally, we also investigated possible relationships between glycemic control/variability and glucose homeostasis (insulin sensitivity/resistance and insulin secretion/beta-cell function). We found a marked relationship between some parameters of glycemic variability and the total insulin secretion, and this was the first study reporting this information in hemodialysis patients. This suggests that in such patients a glucose tolerance test may be complementary to glycemia assessment to get a comprehensive picture of the glucometabolic condition.

## Data Availability Statement

The datasets presented in this article are not readily available because data cannot be made available due to institutional regulations. Requests to access the datasets should be directed to Emanuele Mambelli, Nephrology and Dialysis, Bufalini Hospital, AUSL Romagna, Cesena, Italy (emanuele.mambelli@auslromagna.it).

## Ethics Statement

The studies involving human participants were reviewed and approved by Comitato Etico AUSL Romagna. The patients/participants provided their written informed consent to participate in this study.

## Author Contributions

EM and AT designed the study. EM, SC, and GM conducted the study. AT performed the data analysis. CG supervised the statistical analysis. EM and GM supervised the whole analysis. AT wrote the manuscript. EM, SC, GM, and CG reviewed the manuscript. All authors approved the final version of the manuscript. All authors contributed to the article and approved the submitted version.

## Conflict of Interest

The authors declare that the research was conducted in the absence of any commercial or financial relationships that could be construed as a potential conflict of interest.

## Publisher's Note

All claims expressed in this article are solely those of the authors and do not necessarily represent those of their affiliated organizations, or those of the publisher, the editors and the reviewers. Any product that may be evaluated in this article, or claim that may be made by its manufacturer, is not guaranteed or endorsed by the publisher.

## References

[1] Heinemann L Freckmann G. CGM versus FGM; or, continuous glucose monitoring is not flash glucose monitoring. J Diabetes Sci Technol. (2015) 9:947–50. 10.1177/1932296815603528 26330484 PMC4667350

[2] Bailey T Bode BW Christiansen MP Klaff LJ Alva S. The performance and usability of a factory-calibrated flash glucose monitoring system. Diabetes Technol Ther. (2015) 17:787–94. 10.1089/dia.2014.0378 26171659 PMC4649725

[3] Hoss U Budiman ES. Factory-calibrated continuous glucose sensors: the science behind the technology. Diabetes Technol Ther. (2017) 19:S44–50. 10.1089/dia.2017.0025 28541139 PMC5444502

[4] Gordon I Rutherford C Makarounas-Kirchmann K Kirchmann M. Meta-analysis of average change in laboratory-measured HbA1c among people with type 1 diabetes mellitus using the 14 day flash glucose monitoring system. Diabetes Res Clin Pract. (2020) 164:108158. 10.1016/j.diabres.2020.108158 32333970

[5] Bianchi C Aragona M Rodia C Baronti W de Gennaro G Bertolotto A. Freestyle Libre trend arrows for the management of adults with insulin-treated diabetes: A practical approach. J Diabetes Complications. (2019) 33:6–12. 10.1016/j.jdiacomp.2018.10.012 30446477

[6] Leelarathna L Wilmot EG. Flash forward: a review of flash glucose monitoring. Diabet Med. (2018) 35:472–82. 10.1111/dme.13584 29356072

[7] Padmanabhan A Velayudham B Vijaykumar N Allauddin S. Evaluation of glycemic status during the days of hemodialysis using dialysis solutions with and without glucose. Saudi J Kidney Dis Transpl. (2018) 29:1021–7. 10.4103/1319-2442.243951 30381496

[8] Javherani RS Purandare VB Bhatt AA Kumaran SS Sayyad MG Unnikrishnan AG. Flash glucose monitoring in subjects with diabetes on hemodialysis: a pilot study. Indian J Endocrinol Metab. (2018) 22:848–51. 10.4103/ijem.IJEM_520_18 30766829 PMC6330844

[9] Yajima T Takahashi H Yasuda K. Comparison of Interstitial fluid glucose levels obtained by continuous glucose monitoring and flash glucose monitoring in patients with type 2 diabetes mellitus undergoing hemodialysis. J Diabetes Sci Technol. (2019) 14:1088–94. 10.1177/1932296819882690 31625413 PMC7645125

[10] Jin YP Su XF Yin GP Xu XH Lou JZ Chen JJ. Blood glucose fluctuations in hemodialysis patients with end stage diabetic nephropathy. J Diabetes Complications. (2015) 29:395–9. 10.1016/j.jdiacomp.2014.12.015 25681043

[11] Mirani M Berra C Finazzi S Calvetta A Radaelli MG Favareto F. Inter-day glycemic variability assessed by continuous glucose monitoring in insulin-treated type 2 diabetes patients on hemodialysis. Diabetes Technol Ther. (2010) 12:749–53. 10.1089/dia.2010.0052 20809678

[12] Jung HS Kim HI Kim MJ Yoon JW Ahn HY Cho YM. Analysis of hemodialysis-associated hypoglycemia in patients with type 2 diabetes using a continuous glucose monitoring system. Diabetes Technol Ther. (2010) 12:801–7. 10.1089/dia.2010.0067 20809681

[13] Senda M Ogawa S Nako K Okamura M Sakamoto T Ito S. The strong relation between post-hemodialysis blood methylglyoxal levels and post-hemodialysis blood glucose concentration rise. Clin Exp Nephrol. (2015) 19:527–33. 10.1007/s10157-014-1018-6 25139482

[14] Terawaki Y Nomiyama T Akehi Y Takenoshita H Nagaishi R Tsutsumi Y. The efficacy of incretin therapy in patients with type 2 diabetes undergoing hemodialysis. Diabetol Metab Syndr. (2013) 5:10. 10.1186/1758-5996-5-10 23445717 PMC3598214

[15] Osonoi T Saito M Tamasawa A Ishida H Tsujino D Nishimura R. Effect of hemodialysis on plasma glucose profile and plasma level of liraglutide in patients with type 2 diabetes mellitus and end-stage renal disease: a pilot study. PLoS ONE. (2014) 9:e113468. 10.1371/journal.pone.0113468 25526642 PMC4272272

[16] Yajima T Yajima K Hayashi M Takahashi H Yasuda K. Improved glycemic control with once-weekly dulaglutide in addition to insulin therapy in type 2 diabetes mellitus patients on hemodialysis evaluated by continuous glucose monitoring. J Diabetes Complications. (2018) 32:310–5. 10.1016/j.jdiacomp.2017.12.005 29366733

[17] Abe M Kalantar-Zadeh K. Haemodialysis-induced hypoglycaemia and glycaemic disarrays. Nat Rev Nephrol. (2015) 11:302–13. 10.1038/nrneph.2015.38 25848881 PMC6015632

[18] Spasovski G Vanholder R Allolio B Annane D Ball S Bichet D. Clinical practice guideline on diagnosis and treatment of hyponatraemia. Nephrol Dial Transplant. (2014) 29(Suppl 2):i1–39. 10.1093/ndt/gfu040 Erratum in: *Nephrol Dial Transplant* (2014) 40:924. 24569496

[19] Korsatko S Ellmerer M Schaupp L Mader JK Smolle KH Tiran B. Hypoglycaemic coma due to falsely high point-of-care glucose measurements in an ICU-patient with peritoneal dialysis: a critical incidence report. Intensive Care Med. (2009) 35:571–2. 10.1007/s00134-008-1362-7 19057894

[20] Monnier L Colette C. Glycemic variability: should we and can we prevent it? Diabetes Care. (2008) 31(Suppl 2):S150–4. 10.2337/dc08-s241 18227477

[21] Rodbard D. Interpretation of continuous glucose monitoring data: glycemic variability and quality of glycemic control. Diabetes Technol Ther. (2009) 11(Suppl 1):S55–67. 10.1089/dia.2008.0132 19469679

[22] Rodbard D. New and improved methods to characterize glycemic variability using continuous glucose monitoring. Diabetes Technol Ther. (2009) 11:551–65. 10.1089/dia.2009.0015 19764834

[23] Mari A Tura A Gastaldelli A Ferrannini E. Assessing insulin secretion by modeling multiple meal tests: role of potentiation. Diabetes. (2002) 51:S221–6. 10.2337/diabetes.51.2007.S221 11815483

[24] Tura A Muscelli E Gastaldelli A Ferrannini E Mari A. Altered pattern of the incretin effect as assessed by modelling in individuals with glucose tolerance ranging from normal to diabetic. Diabetologia. (2014) 57:1199–203. 10.1007/s00125-014-3219-7 24658843

[25] Matthews DR Hosker JP Rudenski AS Naylor BA Treacher DF Turner RC. Homeostasis model assessment: insulin resistance and beta-cell function from fasting plasma glucose and insulin concentrations in man. Diabetologia. (1985) 28:412–9. 10.1007/BF00280883 3899825

[26] Tura A Chemello G Szendroedi J Göbl C Færch K Vrbíková J. Prediction of clamp-derived insulin sensitivity from the oral glucose insulin sensitivity index. Diabetologia. (2018) 61:1135–41. 10.1007/s00125-018-4568-4 29484470

[27] Cengiz E Tamborlane WV. A tale of two compartments: interstitial versus blood glucose monitoring. Diabetes Technol Ther. (2009) 11(Suppl 1):S11–6. 10.1089/dia.2009.0002 19469670 PMC2903977

[28] Donauer J Böhler J. Rationale for the use of blood volume and temperature control devices during hemodialysis. Kidney Blood Press Res. (2003) 26:82–9. 10.1159/000070988 12771531

[29] Kooman JP van der Sande FM. Body fluids in end-stage renal disease: statics and dynamics. Blood Purif. (2019) 47:223–9. 10.1159/000494583 30517920 PMC6492508

[30] Clarke WL Cox D Gonder-Frederick LA Carter W Pohl SL. Evaluating clinical accuracy of systems for self-monitoring of blood glucose. Diabetes Care. (1987) 10:622–8. 10.2337/diacare.10.5.622 3677983

[31] Parkes JL Slatin SL Pardo S. Ginsberg, BH. A new consensus error grid to evaluate the clinical significance of inaccuracies in the measurement of blood glucose. Diabetes Care. (2000) 23:1143–8. 10.2337/diacare.23.8.1143 10937512

[32] Ginsberg BH. Factors Affecting blood glucose monitoring: sources of errors in measurement. J Diabetes Sci Technol. (2009) 3:903–13. 10.1177/193229680900300438 20144340 PMC2769960

[33] Qureshi K Clements RH Saeed F Abrams GA. Comparative evaluation of whole body and hepatic insulin resistance using indices from oral glucose tolerance test in morbidly obese subjects with nonalcoholic fatty liver disease. J Obes. (2010) 2010:741521. 10.1155/2010/741521 20798875 PMC2925212

[34] Si Y Shen Y Lu J Ma X Zhang L Mo Y. Impact of acute-phase insulin secretion on glycemic variability in insulin-treated patients with type 2 diabetes. Endocrine. (2020) 68:116–23. 10.1007/s12020-020-02201-y 32006292

[35] Takai M Anno T Kawasaki F Kimura T Hirukawa H Mune T. Association of the glycemic fluctuation as well as glycemic control with the pancreatic β-cell function in Japanese subjects with type 2 diabetes mellitus. Intern Med. (2019) 58:167–73. 10.2169/internalmedicine.1053-18 30146574 PMC6378157

[36] Kohnert KD Heinke P Vogt L Augstein P Salzsieder E. Declining ß-cell function is associated with the lack of long-range negative correlation in glucose dynamics and increased glycemic variability: a retrospective analysis in patients with type 2 diabetes. J Clin Transl Endocrinol. (2014) 1:192–9. 10.1016/j.jcte.2014.09.003 29159101 PMC5685022

[37] Chen T Xu F Su JB Wang XQ Chen JF Wu G. Glycemic variability in relation to oral disposition index in the subjects with different stages of glucose tolerance. Diabetol Metab Syndr. (2013) 5:38. 10.1186/1758-5996-5-38 23876034 PMC3728076

[38] Kohnert KD Augstein P Zander E Heinke P Peterson K Freyse EJ. Glycemic variability correlates strongly with postprandial beta-cell dysfunction in a segment of type 2 diabetic patients using oral hypoglycemic agents. Diabetes Care. (2009) 32:1058–62. 10.2337/dc08-1956 19244086 PMC2681045

[39] Matoba K Hayashi A Shimizu N Moriguchi I Kobayashi N Shichiri M. Comparison of accuracy between flash glucose monitoring and continuous glucose monitoring in patients with type 2 diabetes mellitus undergoing hemodialysis. J Diabetes Complications. (2020) 34:107680. 10.1016/j.jdiacomp.2020.107680 32736927

[40] Ushigome E Matsusaki S Watanabe N Hashimoto T Nakamura N Fukui M. Critical discrepancy in blood glucose control levels evaluated by glycated albumin and estimated hemoglobin A1c levels determined from a flash continuous glucose monitoring system in patients with type 2 diabetes on hemodialysis. J Diabetes Investig. (2020) 11:1570–4. 10.1111/jdi.13286 32356596 PMC7610128

[41] Sangill M Pedersen EB. The effect of glucose added to the dialysis fluid on blood pressure, blood glucose, and quality of life in hemodialysis patients: a placebo-controlled crossover study. Am J Kidney Dis. (2006) 47:636–43. 10.1053/j.ajkd.2006.01.007 16564941

[42] Williams ME Garg R Wang W Lacson R Maddux F Lacson E Jr. High Hemoglobin A1c levels and glycemic variability increase risk of severe hypoglycemia in diabetic hemodialysis patients. Hemodial Int. (2014) 18:423–32. 10.1111/hdi.12110 24274900

[43] Hayashi A Takano K Masaki T Yoshino S Ogawa A Shichiri M. Distinct biomarker roles for HbA1c and glycated albumin in patients with type 2 diabetes on hemodialysis. J Diabetes Complications. (2016) 30:1494–9. 10.1016/j.jdiacomp.2016.08.015 27614726

[44] Gentile S Strollo F Satta E Della-Corte T Romano C Guarino G. Insulin-induced lypodistrophy in hemodialyzed patients: a new challenge for nephrologists? Diabetes Metab Syndr. (2019) 13:3081–4. 10.1016/j.dsx.2019.11.005 31765982

[45] Gentile S Strollo F Satta E Della Corte T Romano C Guarino G. Insulin-related lipohypertrophy in hemodialyzed diabetic people: a multicenter observational study and a methodological approach. Diabetes Ther. (2019) 10:1423–33. 10.1007/s13300-019-0650-2 31222593 PMC6612327

[46] Shi C Liu S Yu HF Han B. Glycemic variability and all-cause mortality in patients with diabetes receiving hemodialysis: a prospective cohort study. J Diabetes Complications. (2020) 34:107549. 10.1016/j.jdiacomp.2020.107549 32033851

[47] Werzowa J Pacini G Hecking M Fidler C Haidinger M Brath H. Comparison of glycemic control and variability in patients with type 2 and posttransplantation diabetes mellitus. J Diabetes Complications. (2015) 29:1211–6. 10.1016/j.jdiacomp.2015.07.014 26264400

[48] Tura A Farngren J Schweizer A Foley JE Pacini G Ahrén B. Four points pre-prandial self-monitoring of blood glucose for the assessment of glycemic control and variability in patients with type 2 diabetes treated with insulin and vildagliptin. Int J Endocrinol. (2015) 2015:484231. 10.1155/2015/484231 26587020 PMC4637474

[49] Frid A Tura A Pacini G Ridderstråle M. Effect of oral pre-meal administration of betaglucans on glycaemic control and variability in subjects with type 1 diabetes. Nutrients. (2017) 9:1004. 10.3390/nu9091004 28895878 PMC5622764

[50] American Diabetes Association. 2. Classification and diagnosis of diabetes: standards of medical care in diabetes-2021. Diabetes Care. (2021) 44(Suppl 1):S15–33. 10.2337/dc21-S002 33298413

[51] Van Cauter E Mestrez F Sturis J Polonsky KS. Estimation of insulin secretion rates from C-peptide levels. Comparison of individual and standard kinetic parameters for C-peptide clearance. Diabetes. (1992) 41:368–77. 10.2337/diab.41.3.368 1551497

[52] Rabkin R Ryan MP Duckworth WC. The renal metabolism of insulin. Diabetologia. (1984) 27:351–7. 10.1007/BF00304849 6389240

[53] Sharma R Rosner MH. Glucose in the dialysate: historical perspective and possible implications? Hemodial Int. (2008) 12:221–6. 10.1111/j.1542-4758.2008.00256.x 18394054

[54] Vlassara H Palace MR. Diabetes and advanced glycation endproducts. J Intern Med. (2002) 251:87–101. 10.1046/j.1365-2796.2002.00932.x 11905595

[55] Kaysen GA. The microinflammatory state in uremia: causes and potential consequences. J Am Soc Nephrol. (2001) 12:1549–57. 10.1681/ASN.V1271549 11423586

[56] Cui L Meng Y Xu D Feng Y Chen G Hu B. Analysis of the metabolic properties of maintenance hemodialysis patients with glucose-added dialysis based on high performance liquid chromatography quadrupole time-of-flight mass spectrometry. Ther Clin Risk Manag. (2013) 9:417–25. 10.2147/TCRM.S49634 24194643 PMC3814896

